# Associations between repetitive head impact exposure and midlife mental health wellbeing in former amateur athletes

**DOI:** 10.3389/fpsyt.2024.1383614

**Published:** 2024-05-28

**Authors:** Claire V. Buddenbaum, Grace O. Recht, Adriana K. Rodriguez, Sharlene D. Newman, Keisuke Kawata

**Affiliations:** ^1^ Department of Kinesiology, Indiana University School of Public Health-Bloomington, Bloomington, IN, United States; ^2^ Alabama Life Research Institute, University of Alabama, Tuscaloosa, AL, United States; ^3^ Program in Neuroscience, The College of Arts and Sciences, Indiana University, Bloomington, IN, United States; ^4^ Department of Pediatrics, Indiana University School of Medicine, Indianapolis, IN, United States

**Keywords:** PTSD, traumatic brain injury, concussion, subconcussive head impacts, depression, ADHD, chronic traumatic encephalopathy

## Abstract

**Introduction:**

Repetitive head impacts (RHI) have been suggested to increase the risk of developing a neurodegenerative disease, and many of these individuals develop a preceding mental health diagnosis. Given the lack of studies among amateur athletes, this study aimed to examine mental health outcomes in middle-aged amateur athletes who have been exposed to RHI through contact sport participation.

**Methods:**

This is a single site, cohort study involving former amateur athletes aged between 30 and 60 with at least 10 years of organized contact or non-contact sport participation. All participants completed demographic and mental health questionnaires. Mental health outcomes included symptoms related to depression, anxiety, post-traumatic stress disorder (PTSD), attention deficit hyperactive disorder (ADHD), and aggression. Self-reported data on mental health diagnoses and associated prescription were elicited and used to estimate odds ratios (OR).

**Results:**

Data from 41 contact athletes and 22 age/sex-matched non-contact athletes were available for analysis. The contact group exhibited a 2.25-fold higher likelihood of being diagnosed with mental health disorders and 1.29-fold higher likelihood of using associated medications compared to the non-contact group. The contact group reported significantly higher PTSD-related symptoms [4.61 (0.03,9.2), p=0.05] compared to the non-contact control group. While not statistically significant, the contact group showed increased depressive [2.37 (0.05, 4.79), p=0.07] and ADHD symptoms [4.53 (0.51, 9.57), p=0.08] compared to controls. In a secondary analysis, a distinct trend emerged within the contact group, revealing pronounced elevations in mental health symptoms among individuals with lower socioeconomic status (<$50,000/year) compared to higher income subgroups, and these symptoms decreased as income levels rose [depression: -3.08 (-4.47, -1.7), p<0.001; anxiety: -1.95 (-3.15, -0.76), p=0.002; ADHD: -4.99 (-8.28, -1.69), p=0.004; PTSD: -4.42 (-7.28, -1.57), p=0.003; aggression: -6.19 (-11.02, -1.36), p=0.01]. This trend was absent in the non-contact control group.

**Discussion:**

Our data suggest that even individuals at the amateur level of contact sports have an increased likelihood of being diagnosed with mental health disorders or experiencing mental health symptoms compared to non-contact athletes. Our findings indicate that socioeconomic status may have an interactive effect on individuals’ mental health, particularly among those with a long history of RHI exposure.

## Introduction

Over 2 million male and female athletes compete in high school and college contact sports annually (1), exposing themselves to repetitive head impacts (RHI) (2, 3). These concussive and subconcussive head impacts pose a potential threat, capable of triggering cognitive dysfunction, neuropsychiatric symptoms, and the potential development of neurodegenerative conditions, such as Alzheimer’s disease related dementia, including chronic traumatic encephalopathy (CTE) (4, 5). Recent findings indicate that declines in mental health may precede cognitive deterioration and dementia diagnoses. Notably, individuals with CTE often exhibit significant changes in mental health conditions during their 40s and 50s (6–8). Despite this knowledge, the inquiry of the interactive effects of mental health and RHI is still in its early stages, with a predominant focus on male professional athletes, particularly in football (4, 9). Given the larger population of amateur athletes susceptible to mental health declines, it becomes imperative to characterize the midlife mental health integrity in those who participated in RHI-prone contact sports at the amateur level for both males and females.

In the past decade, as societal awareness and acceptance for mental health conditions have gained traction, a strong association between mental health and brain injury has surfaced. For example, not only the meta-analysis by Gornall et al. (10), but also the largest retrospective cohort study underscored this association, revealing a 1.4-fold increase in the incidence of mental health issues, including anxiety, neurotic, and mood disorders, among children and adolescents with concussions (n=152,321) compared to orthopedic injury controls (11). A notable finding also includes the vulnerability of female adolescents to internalizing issues, such as depression, anxiety, and withdrawal following a concussion (12). This trend extends in young adults, where individuals with a history of concussion exhibit higher panic symptoms and increased use of alcohol and cannabis, with the relationship between mood disorder and concussion being more pronounced in females than males (13). However, it is noteworthy that a prospective study by Kercher et al. (14) failed to replicate these results in the context of subconcussive RHI. Throughout a single high school football season, depression, anxiety, thriving, and psychological satisfaction scores remained consistent, showing no significant association with head impact exposure as measured by instrumented mouthguards.

In contrast to amateur athletes, considerable research has been dedicated to examining the effects of RHI on professional football players. An earlier study involving 42 retired National Football League (NFL) players ages 41–77 found a significant elevation in depressive symptoms among these retired players compared to those who had never sustained concussions or played football (15). Subsequent studies have further illuminated cases where prolonged exposure to RHI may contribute to profound and rapid alterations in impulse control, aggression, depression, and anxiety symptoms later in life (4, 7, 16–19). However, it is crucial to acknowledge a critical limitation in many of these post-mortem studies: mental health conditions are often reported by informants rather than the athlete themselves. Thus, there is a pressing need for pre-mortem studies, especially among middle-aged adults, to explore the potential association between RHI and mental health symptoms.

The purpose of this present study is to examine the impacts of lifetime exposure to RHI on mental health outcomes in middle-aged, amateur athletes. Given that depression, anxiety, impulsivity, PTSD, and aggression consistently manifest as clinical symptoms of CTE (4, 7, 8), this study is specifically tailored to explore these mental health outcomes. We hypothesized that individuals engaged in contact sports would exhibit worse mental health outcomes (increased depression, anxiety, attention, and aggression symptomology) than their counterparts who participated in non-contact sports. Recognizing the potential influence of the duration of sport experience and socioeconomic status on mental health symptoms (20), we conducted a secondary analysis to delve into the effects of these factors on mental health outcomes in both contact and non-contact control groups.

## Materials and methods

### Participants

**Figure 1:**
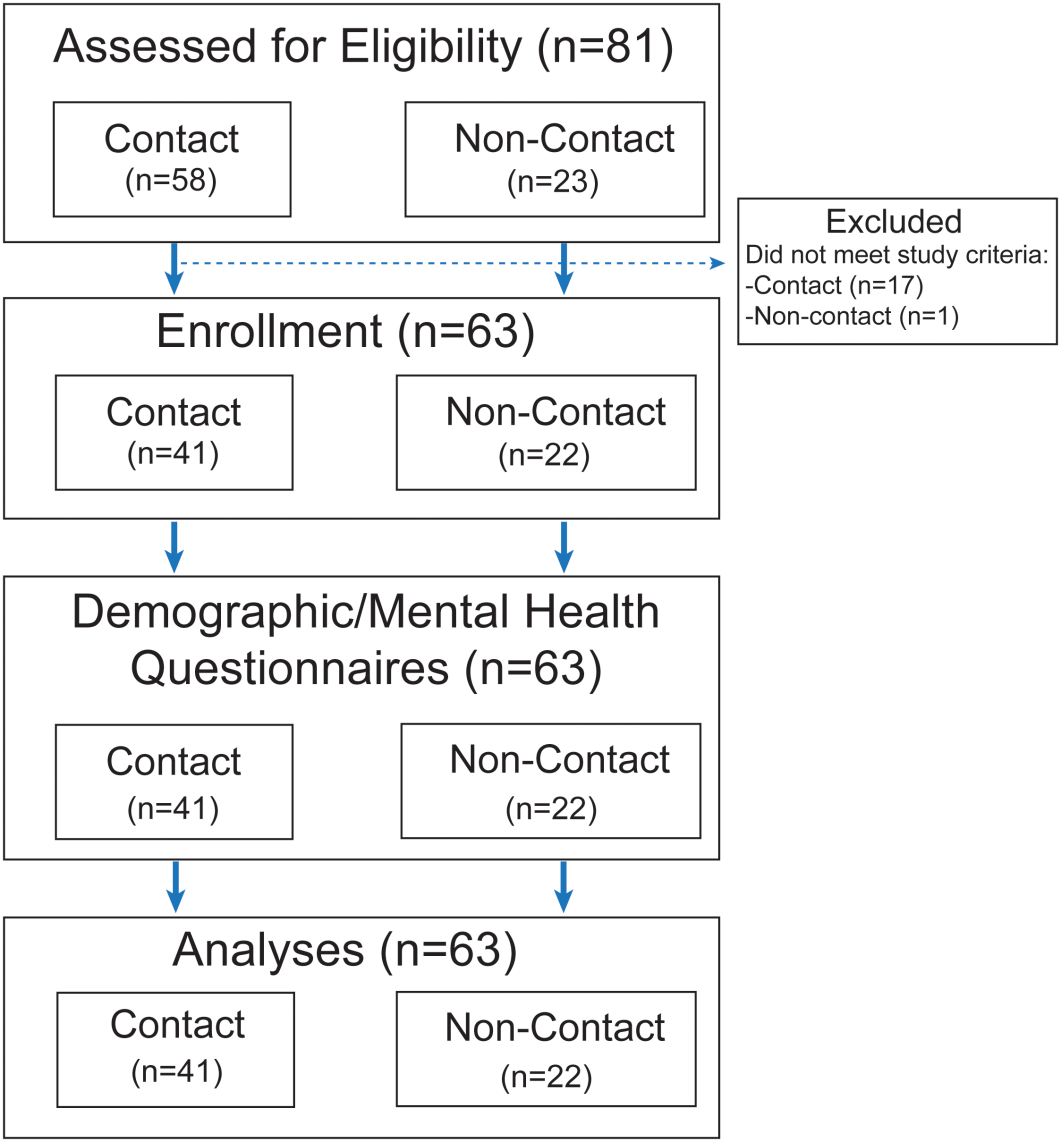
Study flow chart.

This cohort study included 63 participants, including 41 contact sport athletes (32 males, 9 females) and 22 age- and sex-matched non-contact athletes (14 males, 8 females). Potential participants were recruited by emails to community partners, social media posts, and Indiana CTSI’s iCONNECT. Data was collected from February to September 2023. Inclusion criteria for the contact group included having at least 10 years of organized contact sport participation experience and being between the ages of 30 and 60. For the non-contact group, participants needed to have at least 10 years of participation in organized non-contact sports, no history of participation in contact sports, and be between the ages of 30 and 60. Exclusion criteria for both groups were any head, neck, or facial injuries, including concussions, in the 6 months prior to study participation, pregnancy, a history of any neurological disorders, impaired decisional capacity, metal implants in the head, and any implanted electro/magnetic devices. **Figure 1** describes the flow of this study. All participants provided informed consent prior to participation in any study procedures. The study protocol was approved by the Indiana University Institutional Review Board (#17763).

### Measures

Demographic questionnaires collected information, such as age, sex, race, ethnicity, concussion history, duration of sports experience, types and duration of each sport played, and sociodemographic characteristics. Participants were instructed to complete the following mental health questionnaires.

#### Patient Health Questionnaire – 9

The PHQ-9 is a self-administered questionnaire that assesses depressive symptoms. The PHQ-9 consists of 9 questions with a 4-point Likert scale for responses. The 9 questions assess the DSM-5 diagnostic criteria with 0 being not at all, 1 being several days, 2 being more than half days, and 3 being nearly every day. Higher scores represent worse depressive symptomology. If a participant scored a 2 or higher on 5 of the 9 criteria, it is considered major depression (21).

#### General Anxiety Disorder-7

The GAD-7 is a self-administered questionnaire that assesses the presence of generalized anxiety disorder. The GAD-7 consists of 7 questions with a 4-point Likert scale for responses. The 7 questions assess the DSM-5 diagnostic criteria for generalized anxiety disorder with 0 being not at all, 1 being several days, 2 being more than half of days, and 3 being nearly every day. A score of 0 to 4 is minimal anxiety, 5–9 is mild anxiety, 10–14 is moderate anxiety, and above 15 is severe anxiety (22).

#### DSM-5 diagnostic criteria for ADHD

The DSM-5 Diagnostic Criteria for ADHD is an 18-item self-report measure that corresponds to the key symptoms of an ADHD diagnosis in adults (23). Participants were to respond to the 18-items with how they have conducted themselves over the past 6 months. It is a 4-point Likert scale ranging from 0 “Never” to 3 “Very Often”. To meet the DSM-5 criteria for a diagnosis of ADHD, the participant would have to have 5 responses of “Often” (2) or “Very Often” to either the first 9 inattentive items (1–9) or the 9 (10–18) hyperactive-impulsive items.

#### PTSD Checklist – Civilian Version

The PCL-C is a 17-item, self-report measure that corresponds to the key symptoms of PTSD (24). Participants were to respond to the 17 items with how much they have been bothered by certain symptoms in the past month using a 5-point Likert Scale. Responses range from 1 “Not at all” to 5 “Extremely”. A total score of the PCL-C score was used in the analyses.

#### Aggression Questionnaire

The AQ is a 34-item self-report scale to assess the 4 components of aggression: physical aggression, verbal aggression, anger, and hostility. Participants were instructed to respond to each question with how they interact with other people. The responses are a 5-point Likert scale ranging from 1 “not at all like me” to 5 “completely like me”. All responses are summed at the end for a total aggression score (25, 26).

### Statistical analysis

Differences in demographic variables between the contact and non-contact groups were assessed by independent samples t-tests for continuous variables and chi-square for categorical variables. Odds ratios (OR) and the associated 95% confidence intervals (CI) were estimated for the potential group differences in the likelihood of mental health diagnoses and the associated prescriptions. Multivariable linear regression models were used to examine group differences in mental health outcomes, with the PHQ-9, GAD-7, DSM-5 ADHD, PCL-C, and AQ being set as the primary outcomes. The models were adjusted by covariates, including age, sex, and education level. The significance level was set at p<0.05.

Secondly, a linear regression model was used to examine relationships between mental health outcomes and the cumulative duration in years of contact sports (for the contact group) and non-contact sports (for non-contact sports) in each group. The model was adjusted by covariates, including age, sex, and education level. Finally, participants in both groups were categorized into 4 sub-groups based on their income levels: ≤$50k, $51k–$100k, $101k–$150k, or $151k≤. Generalized linear models were used to estimate the effects of group (contact, non-contact), income level (4 subgroups), and group-by-income interactions on mental health outcomes, with age, sex, and education as covariates. If significant interaction effects were observed, we conducted *post-hoc* analyses with Bonferroni corrections to examine at what income levels group difference emerged, and the level of significance was set at p<0.0125 to reflect 4 income sub-groups. All analyses were conducted using R, version 4.2.1 (R Project for Statistical Computing) with the nlme package. The analysis was summarized by providing a contrast estimate with its 95% CI and a p-value in the following format: [estimate (CI_low, CI_high); p-value].

## Results

### Demographics

A total of 63 participants were included in the study (contact n=41, non-contact n=22). Of the 41 participants in the contact group (age 42.2 ± 9.4 years), 32 (78%) were male. Of the 22 participants in the non-contact group (age 44.8 ± 8.4 years), 14 (63.6%) were male. Participants in both the contact and non-contact group were predominately White (92.7% - 95.5%). The contact group had 15.1 (5.0) years of contact sport experience and the non-contact group had 16.8 (5.9) years of non-contact sport experience. Three participants (n=1 contact; n=2 non-contact) were excluded due to no response to mental health questionnaires. **Figure 1** depicts the study flow chart. Demographics are summarized in **Table 1**.

**Table 1:** Group demographics.

Group	Contact Sport	Non-Contact Sport	p-value
n	41	22	–
Sex (%)	32 M (78%)	14 M (63.6%)	0.35
Age, y	42.2 (9.4)	44.8 (8.4)	0.24
**No. of previous concussion**			0.42
0, *n* (%)	26 (63.4)	19 (86.3)	-
1, *n* (%)	6 (14.6)	1 (4.5)	–
2, *n* (%)	2 (4.9)	1 (4.5)	–
3+, *n* (%)	7 (17.1)	1 (4.5)	–
Organized Sport Experience^*#,^ *n* (%)	15.1 (5.0)	16.8 (5.9)	0.26
Football	24 (63.2)	0	–
Soccer	16 (42.1)	0	–
Wrestling	9 (23.7)	0	–
Hockey	7 (18.4)	0	–
Baseball	0	15 (68.2)	–
Cross Country/Track	0	8 (36.4)	–
Volleyball	0	6(27.3)	–
Tennis	0	4 (18.2)	–
**Race, *n* (%)**			0.77
White	38 (92.7)	21 (95.5)	–
Black/African American	0 (0)	0 (0)	-
Asian	2 (4.9)	1 (4.5)	–
Multiracial	1 (2.4)	0 (0)	–
**Ethnicity, *n* (%)**			0.23
Not Latino/Hispanic	38 (100)	20 (90.9)	-
Latino/Hispanic	0 (0)	2 (9.1)	-
Mental Health Diagnosis, *n (%)* **	8 (20)	2 (10)	0.171
Mental Health Related Medications, *n (%)* **	5 (13.5)	2 (10)	0.463
Annual Income, *n* (%)			
$50k and under	10 (24.4)	4 (18.2)	–
$51k - $100k	14 (34.1)	7 (31.8)	–
$101k - $150k	12 (29.3)	4 (18.2)	–
$151k +	5 (12.2)	7 (31.8)	–

*The four most participated in sports.

^#^N and percentages are equal to more than 100% due to subjects participating in multiple sports.

**: n=1 in the contact group and n=2 in the control group reported prefer not to answer.

### Mental health diagnosis and prescription between the contact and non-contact groups

Self-reported mental health diagnosis and associated prescriptions were elicited as part of demographics. The contact group was 2.25 times more likely to be diagnosed with mental health disorders [OR=2.25 (0.5, 16.0)] and 1.29 times more likely to be taking associated medications [OR=1.29, (0.25, 9.6)] relative to the non-contact group.

### Mental health-related symptoms between the contact and non-contact groups

**Figure 2:**
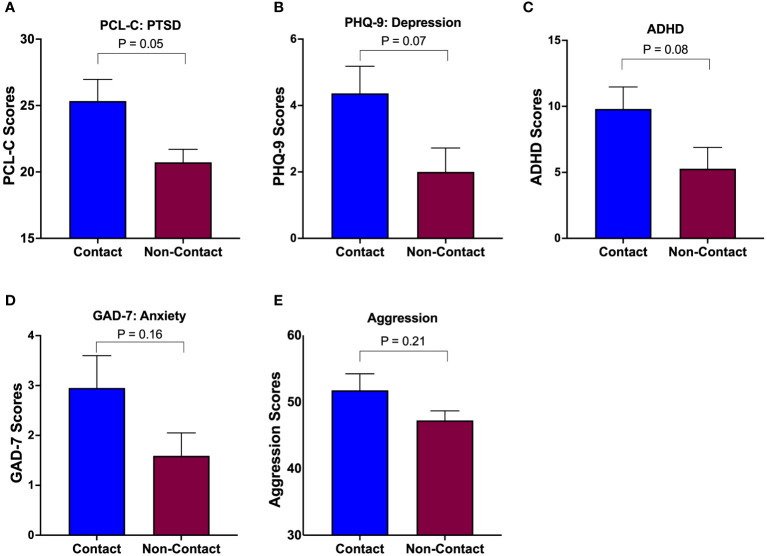
Group differences in mental health symptoms. The contact group exhibited elevated levels of symptoms in all aspects of mental health (PTSD **(A)**, Depression **(B)**, ADHD **(C)**, Anxiety **(D)**, and Aggression **(E)**) compared to the non-contact control group, but statistical significance was limited to PTSD **(A)**.

Independent samples t-tests showed that the contact group had significantly elevated symptoms in PCL-C (p=0.018), PHQ-9 (p=0.034), and ADHD (p=0.05). However, our regression model including covariates failed to detect group differences in PHQ-9 and ADHD, due to age being a significant covariate modulating the outcomes. Nonetheless, the statistically significant group difference was retained in PTSD symptoms (via PCL-C), where the contact group scored 4.61 points higher (worse) on the PCL-C [4.61 (0.03,9.2), p=0.05] than the non-contact group. No statistically significant group differences were observed in depressive symptoms [PHQ-9: 2.37 (0.05, 4.79), p=0.07], ADHD symptoms [4.53 (0.51, 9.57), p=0.08], aggression symptoms [AQ: 4.5 (2.46, 11.47), p=0.21], and anxiety symptoms [GAD-7: 1.36 (0.50, 3.22), p=0.16: **Figure 2**]. See **Supplementary Table 1** for average values of each outcome.

### Associations between mental health outcome and years of sport experience

There were no significant associations between the cumulative duration in years of sports played and mental health outcomes in both groups. See **Supplementary Figure 2**.

### Socioeconomic status differentially impacted mental health outcomes between groups

**Figure 3:**
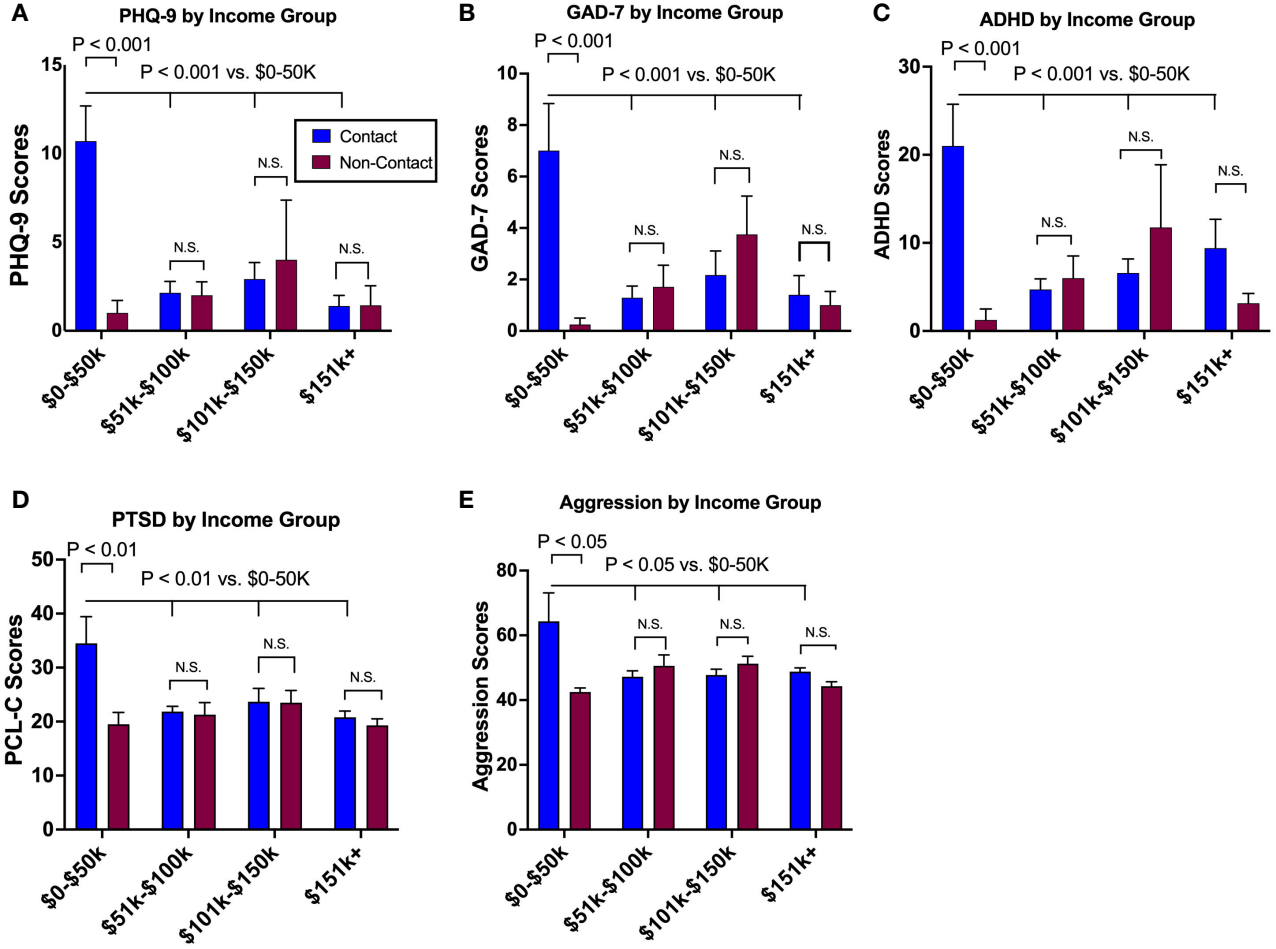
The modulatory effects of socioeconomic status on mental health outcomes. Socioeconomic status, as surrogated by annual income, differentially impacted mental health symptoms between groups, where the lowest tier of annual income (<$50,000) showed the greatest levels of depression **(A)**, anxiety **(B)**, ADHD **(C)**, PTSD **(D)**, and aggression **(E)** as compared to other income tiers in the contact group. Such a tend was absent in the non-contact group. N.S., not significant.

For the PHQ-9, the contact group showed that income level had a significant impact on depression scores [-3.08 (-4.47, -1.7), p<0.001], while the non-contact group did not [0.73 (-2.36, 0.89), p=0.35], which was further illustrated by a significant income x group interaction [3.02 (0.93, 5.10), p=0.004]. Similarly, anxiety, ADHD, PTSD, and aggression scores were impacted by income level for the contact group [GAD-7: -1.95 (-3.15, -0.76), p=0.002; ADHD -4.99 (-8.28, -1.69), p=0.004; PCL-C: -4.42 (-7.28, -1.57), p=0.003; aggression: -6.19 (-11.02, -1.36), p=0.01], but not for the non-contact group. A significant income by group interaction was detected in GAD-7 [1.91 (0.21, 3.61), p=0.03]. Statistical output is detailed in **Table 2**. The follow-up *post-hoc* analyses revealed that group differences in depression, anxiety, ADHD, and PTSD symptoms occurred at the ≤$50k level, where the contact group in this specific income level reported significantly greater symptoms than the non-contact group, but no group differences in any other income levels (**Figure 3**).

**Table 2:** Statistical output reflecting group by income interactions.

	Income				
	PHQ-9	GAD-7	ADHD	PCL-C	AQ
**Contact**	-3.08 -4.47 to -1.7 P < 0.001**	-1.95 -3.15 to –0.76 P = 0.002**	-4.99 -8.28 to –1.69 P = 0.004**	-4.42 -7.28 to –1.57 P = 0.003**	-6.19 -11.02 to –1.36 P = 0.01*
**Non-Contact**	-0.73 -2.35 to 0.89 P = 0.35	-0.32 -1.32 to 0.67 P = 0.50	-0.68 -4.46 to 3.09 P = 0.71	-0.78 -3.18 to 1.62 P = 0.50	-1.38 -4.89 to 2.11 P = 0.41
**Interaction**	3.02 0.93 to 5.10 P = 0.004**	1.91 0.21 to 3.61 P = 0.03*	-4.47 -0.22 to 9.16 P = 0.06	3.92 -0.27 to 8.13 P = 0.07	5.04 -1.48 to 11.56 P = 0.13

β-value, 95% Confidence Interval, p-value.

* = when p is greater than 0.0125 but still technically significant.

** = when p is less than 0.0125.

## Discussion

The current study presents a potential interactions between midlife mental health well-being and engagement in contact sports. Drawing data from a cohort of middle-aged, amateur athletes, the current study yielded three notable findings. First, contact sport athletes were twice as likely to be diagnosed with mental health conditions, and despite some of them taking medications, the contact group had elevated (worse) scores in PTSD, depression, and ADHD symptoms compared to the non-contact group, suggesting an overall decline in mental health outcomes for contact sports athletes. Second, the absence of a significant correlation between mental health symptoms and the number of years participated in contact sports may be attributed to the limitation of this variable in capturing cumulative effects of RHI exposure over time. Lastly, among contact sport athletes, individuals with a lower income level, particularly less than $50k a year, experienced a significantly detrimental effect on symptoms related to depression, anxiety, ADHD, and PTSD symptoms, whereas no such impact was observed in non-contact athletes. Overall, these data suggest that a decade or more of participation in contact sports may be associated with adverse effects on mental health during midlife.

Concussions are widely acknowledged for their potential to trigger an array of mental health symptoms, including anxiety, sadness, loneliness, and irritability (3, 11, 13). During 2014 to 2018, a large-scale cohort study conducted by the TRACK-TBI consortium employed identical mental health screening tools, such as PLC and PHQ-9, as the current study and revealed that at the 3-month follow-up, 18.7% and 8.8% of concussed patients manifested PTSD and major depressive disorder, respectively. In contrast, only 7.6% and 3.0% of orthopedic trauma controls developed these conditions (27). Moreover, a survey involving retired NFL players disclosed that 11% of them reported clinical depression diagnosis, which is especially notable among individuals with previous history of concussions (28). These findings were consistently validated in a series of studies in retired professional contact sports athletes (29–31). Our own observations align with these previous findings, demonstrating that the contact group exhibited heightened PTSD and depression symptoms and were two times more likely to be diagnosed with mental health conditions to the control group.

It is worth noting that despite well-established connections between concussions and mental health challenges, there is a scarcity of data regarding subconcussive RHI. For example, a longitudinal study conducted in 2023 by Kercher et al. (14) reported that mental health symptoms related to depression, anxiety, and motivation remained unaffected post-season among adolescent football players, even among those who experienced 100+ head hits and those who started playing tackle football in their youth. One line of research reinforces the notion that participating in tackle football during adolescence does not correlate with negative outcomes in depression, anxiety, alcohol use, and suicidality during young adulthood (32–34). However, opposing research suggests that despite the absence of clinically discernible psychiatric symptoms, adolescent football players displayed alterations in cortical morphology and neural activation patterns in brain regions vital for mental well-being, including the cingulate cortex, precuneus, and prefrontal cortex (9). A study published in 2023 by McKee et al. reported that in a cohort of brain donors all under the age of 30, with extensive RHI history, 70% were reported to have depressive symptoms, and 56.8% had neurobehavioral dysregulation (4). Similarly, retired amateur rugby players (48.3±11.0 years old) were 2-to-3-times more likely to endorse depression and anxiety symptoms compared to retired non-contact athletes (35). These studies indicate that there may be an emergence of mental health symptoms later on and may persist with age in individuals with RHI history. The complexities surrounding the effects of RHI on mental health are attributed to inherent limitations in subjective reporting and potential significant modulating factors, with socioeconomic status being one such factor.

The interactions among developmental socioeconomic status, brain injury effects, and mental/cognitive health are intertwined and difficult to isolate. On one hand, low socioeconomic status environments during childhood can heighten aggressive traits (36). Meanwhile, pediatric traumatic brain injury (TBI) is more prevalent in lower socioeconomic status, which can also affect mental health wellbeing (37). Furthermore, those with a background of low education and rural childhood residency carry a 6.5 times greater risk of developing Alzheimer’s disease compared to those with high education and urban childhood residency. Nevertheless, a key factor attributing to group differences we observed in mental health outcomes is likely the RHI exposure. Participants in the lowest income tier (<$50k/year) displayed markedly elevated symptoms across all mental health domains, with depression and anxiety symptoms being 5 times greater than other income tiers. Strikingly, this pattern did not manifest in the non-contact control group, challenging the conventional understanding of socioeconomic status as a modulatory factor for mental well-being (38–41). Our observation raises questions about whether RHI exposure disproportionately affects those with lower socioeconomic status or if RHI-induced mental health challenges significantly impact one’s career trajectory. Insights from the recent TRACK-TBI Study suggest that 21% (92 of 435) of patients with concussions experienced a decline in annual income at the 12-month follow-up due to lingering symptoms impeding their work, coupled with a lack of employer assistance. Concussive RHI is not only associated with persisting cognitive and mental symptoms but also entails substantial economic consequences for some patients (42). In more severe cases of TBI, Haines et al. (43) reported a significant association between low socioeconomic status and higher mortality rate, longer hospital stays, and slower discharge from inpatient rehabilitation. Our novel data on the socioeconomic impact on RHI-related mental health challenges play a pivotal role in future longitudinal studies, emphasizing the need to include and extrapolate socioeconomic status considerations when investigating RHI-related mental and neurodegenerative disorders.

### Limitations

There were several limitations in this study. First, it is important to acknowledge that all previous diagnoses and medication information relied on self-reporting, highlighting a potential source of bias. Future research could benefit from incorporating interview-based assessments, such as the Structured Clinical Interview for DSM Disorders (SCID-5) (44), to validate the diagnosis of mental health disorders. To maintain confidentiality and participant comfort during questionnaire responses, we implemented a closed-room environment. However, in the interest of rigorously capturing participants’ mental state, examining mental health well-being at multiple points throughout the day would help mitigate the potential fluctuations in mental health symptoms within a single day. In addition, it would be beneficial to longitudinally track mental health symptoms to establish temporal relationships between RHI history and the onset/trajectory of mental health well-being. This study did not explore the dose-response relationship or differentiate among medication types for individuals managing their mental health. A more extensive sample size would have allowed for a more in-depth analysis of potential medication effects on mental health outcomes. We used non-contact sport athletes as controls to compare the effects of lifetime exposure to contact sports on retired, amateur contact sport athletes. Nonetheless, we could have also included another group of non-athletic controls to account for the potential therapeutic effects of physical activity, given that exercise is known to improve various mental health conditions. Types and doses of mental health-related medication should be examined and accounted for in the future study. Despite efforts to match participants by age and sex across various sports, there is a notable lack of ethnic and racial diversity in the current study. To enhance the generalizability of our findings, future research should aim to include samples from different geographical locations, thereby increasing diversity and expanding the applicability of our results.

### Clinical implications

Participating in sports, especially component of physical activity, is a strong predictor for longevity (45–48). However, our data, together with a growing body of CTE literature, raise a concern that if long history of contact sport participation may not achieve the same level of benefits as non-contact sports. Medical practitioners are encouraged to incorporate the history of contact sports experience and RHI exposure into diagnosis and treatment. Furthermore, it is important to consider the interactive effects of socioeconomic status when treating for individuals with history of RHI.

## Conclusions

Our data suggest that even individuals at the amateur level of contact sports have an increased likelihood to be diagnosed with mental health disorders or experience mental health symptoms, especially depression, later in life compared to non-contact athletes. Income levels played a significant role in modulating mental health symptoms in the contact group but not in the control group, where the lowest tier of income level within the contact group was associated with the highest levels of depression, anxiety, PTSD, ADHD, and aggression symptoms, compared to other income tiers. However, there was no correlation between years of contact sports experience and mental health symptoms, which may indicate the insensitive nature of the variable “years of sports experience” to reflect the actual dosage of RHI experienced throughout one’s career. Taken together, it would be beneficial for mental health professionals to acquire contact sport participation history and view it as a risk factor for mental health disorders. Secondly, it is important to recognize that mental health disorders are most prominent in contact athletes who are in the lower to middle income levels. A future longitudinal study including both males and female amateur athletes is warranted to establish the temporal relationships between RHI exposure and later onset of mental health challenges.

## Data availability statement

The original contributions presented in the study are included in the article/**Supplementary Material**. Further inquiries can be directed to the corresponding author.

## Ethics statement

The studies involving humans were approved by Indiana University Institutional Review Boards. The studies were conducted in accordance with the local legislation and institutional requirements. The participants provided their written informed consent to participate in this study.

## Author contributions

CB: Data curation, Investigation, Methodology, Writing – original draft, Writing – review & editing. GR: Conceptualization, Data curation, Investigation, Methodology, Supervision, Writing – original draft, Writing – review & editing. AR: Data curation, Investigation, Writing – original draft, Writing – review & editing. SN: Conceptualization, Supervision, Writing – original draft, Writing – review & editing. KK: Conceptualization, Funding acquisition, Investigation, Project administration, Supervision, Visualization, Writing – original draft, Writing – review & editing

## References

[1] National Federation of State High School Associations. NFHS Releases First High School Sports Participation Survey in Three Years. The National Federation of State High School Associations (2022). Available at: https://www.nfhs.org/articles/nfhs-releases-first-high-school-sports-participation-survey-in-three-years/.

[2] Bailes JE Petraglia AL Omalu BI Nauman E Talavage T. Role of subconcussion in repetitive mild traumatic brain injury. J Neurosurg. (2013) 119:1235–45. doi: 10.3171/2013.7.JNS121822 23971952

[3] Patricios JS Schneider KJ Dvorak J Ahmed OH Blauwet C Cantu RC. Consensus statement on concussion in sport: the 6th International Conference on Concussion in Sport-Amsterdam, October 2022. Br J Sports Med. (2023) 57:695–711. doi: 10.1136/bjsports-2023-106898 37316210

[4] Mckee AC Mez J Abdolmahammadi B Butler M Huber BR Uretsky M. Neuropathologic and clinical findings in young contact sport athletes exposed to repetitive head impacts. JAMA Neurol. (2023) 80(10):1037–50. doi: 10.1001/jamaneurol.2023.2907 PMC10463175 37639244

[5] Nowinski CJ Bureau SC Buckland ME Curtis MA Daneshvar DH Faull RLM. Applying the Bradford hill criteria for causation to repetitive head impacts and chronic traumatic encephalopathy. Front Neurol. (2022) 13:938163. doi: 10.3389/fneur.2022.938163 35937061 PMC9355594

[6] McKee AC Stern RA Nowinski CJ Stein TD Alvarez VE Daneshvar DH. The spectrum of disease in chronic traumatic encephalopathy. Brain: J Neurol. (2013) 136:43–64. doi: 10.1093/brain/aws307 PMC3624697 23208308

[7] Mez J Daneshvar DH Kiernan PT Abdolmohammadi B Alvarez VE Huber BR. Clinicopathological evaluation of chronic traumatic encephalopathy in players of American football. Jama. (2017) 318:360–70. doi: 10.1001/jama.2017.8334 PMC5807097 28742910

[8] McKee AC Stein TD Huber BR Crary JF Bieniek K Dickson D. Chronic traumatic encephalopathy (CTE): criteria for neuropathological diagnosis and relationship to repetitive head impacts. Acta neuropathol. (2023) 145:371–94. doi: 10.1007/s00401-023-02540-w PMC10020327 36759368

[9] Zuidema TR Hou J Kercher KA Recht GO Sweeny SH Chenchaiah N. Cerebral cortical surface structure and neural activation pattern among adolescent football players. JAMA Netw Open. (2024) 7:e2354235. doi: 10.1001/jamanetworkopen.2023.54235 38300622 PMC10835513

[10] Gornall A Takagi M Morawakage T Liu X Anderson V. Mental health after paediatric concussion: a systematic review and meta-analysis. Br J Sports Med. (2021) 55:1048–58. doi: 10.1136/bjsports-2020-103548 33926965

[11] Ledoux AA Webster RJ Clarke AE Fell DB Knight BD Gardner W. Risk of mental health problems in children and youths following concussion. JAMA Netw Open. (2022) 5:e221235. doi: 10.1001/jamanetworkopen.2022.1235 35254429 PMC8902648

[12] Gornall A Takagi M Clarke C Babl FE Davis GA Dunne K. Behavioral and emotional difficulties after pediatric concussion. J neurotrauma. (2020) 37:163–9. doi: 10.1089/neu.2018.6235 31072265

[13] Newman SD Grantz JG Brooks K Gutierrez A Kawata K. Association between history of concussion and substance use is mediated by mood disorders. J neurotrauma. (2020) 37:146–51. doi: 10.1089/neu.2019.6550 PMC7364309 31359826

[14] Kercher KA Steinfeldt JA Rettke DJ Zuidema TR Walker MJ Martinez Kercher VM. Association between head impact exposure, psychological needs, and indicators of mental health among U.S. High school tackle football players. J Adolesc Health. (2023) 72:502–9. doi: 10.1016/j.jadohealth.2022.11.247 PMC10033334 36610880

[15] Didehbani N Munro Cullum C Mansinghani S Conover H Hart J Jr. Depressive symptoms and concussions in aging retired NFL players. Arch Clin Neuropsychol. (2013) 28:418–24. doi: 10.1093/arclin/act028 PMC4007104 23644673

[16] Gregory H. Making a murderer: Media renderings of brain injury and Aaron Hernandez as a medical and sporting subject. Soc Sci Med. (2020) 244:112598. doi: 10.1016/j.socscimed.2019.112598 31689566 PMC6964160

[17] Omalu BI DeKosky ST Minster RL Kamboh MI Hamilton RL Wecht CH. Chronic traumatic encephalopathy in a National Football League player. Neurosurgery. (2005) 57:128–34; discussion 128–34. doi: 10.1227/01.NEU.0000163407.92769.ED 15987548

[18] Omalu BI DeKosky ST Hamilton RL Minster RL Kamboh I Shakir AM. Chronic traumatic encephalopathy in A national football league player: part II. Neurosurgery. (2006) 59:1086–93. doi: 10.1097/00006123-200605000-00036 17143242

[19] Stern RA Daneshvar DH Baugh CM Seichepine DR Montenigro PH Riley DO. Clinical presentation of chronic traumatic encephalopathy. Neurology. (2013) 81:1122–9. doi: 10.1212/WNL.0b013e3182a55f7f PMC3795597 23966253

[20] Asken BM Sullan MJ Snyder AR Houck ZM Bryant VE Hizel LP. Factors influencing clinical correlates of chronic traumatic encephalopathy (CTE): a review. Neuropsychol Rev. (2016) 26:340–63. doi: 10.1007/s11065-016-9327-z PMC5507554 27561662

[21] Kurt Kroenke M Robert L. Spitzer MD Janet B.W. Williams DSW. The PHQ-9. J Gen Internal Med. (2001) 16:606–13. doi: 10.1046/j.1525-1497.2001.016009606.x PMC1495268 11556941

[22] Spitzer RL Williams JBW Lowe B. A brief measure for assessing generalized anxiety disorder: the GAD-7. JAMA Internal Med. (2006) 166:1092–7. doi: 10.1001/archinte.166.10.1092 16717171

[23] Solanto MV Wasserstein J Marks DJ Mitchell KJ. Diagnosis of ADHD in adults: what is the appropriate DSM-5 symptom threshold for hyperactivity-impulsivity? J Atten Disord. (2012) 16:631–4. doi: 10.1177/1087054711416910 21976031

[24] Conybeare D Behar E Solomon A Newman MG Borkovec TD. The PTSD Checklist-Civilian Version: reliability, validity, and factor structure in a nonclinical sample. J Clin Psychol. (2012) 68:699–713. doi: 10.1002/jclp.21845 22517497

[25] Bus AH Perry M. The aggression questionnaire. Personality processes and individual differences. J Personality Social Psychol. (1992) 63:452–9. doi: 10.1037//0022-3514.63.3.452 1403624

[26] Bryant FB Smith BD. Refining the architecture of aggression: A measurement model for the buss-perry aggression questionnaire. J Res Pers. (2001) 35:138–67. doi: 10.1006/jrpe.2000.2302

[27] Stein MB Jain S Giacino JT Levin H Dikmen S Nelson LD. Risk of posttraumatic stress disorder and major depression in civilian patients after mild traumatic brain injury: A TRACK-TBI study. JAMA Psychiatry. (2019) 76:249–58. doi: 10.1001/jamapsychiatry.2018.4288 PMC6439818 30698636

[28] Guskiewicz KM Marshall SW Bailes J McCrea M Harding HP Jr. Matthews A. Recurrent concussion and risk of depression in retired professional football players. Med Sci Sports Exerc. (2007) 39:903–9. doi: 10.1249/mss.0b013e3180383da5 17545878

[29] Kerr ZY Marshall SW Harding HP Jr. Guskiewicz KM. Nine-year risk of depression diagnosis increases with increasing self-reported concussions in retired professional football players. Am J sports Med. (2012) 40:2206–12. doi: 10.1177/0363546512456193 22922518

[30] Brett BL Kerr ZY Walton SR Chandran A Defreese JD Mannix R. Longitudinal trajectory of depression symptom severity and the influence of concussion history and physical function over a 19-year period among former National Football League (NFL) players: an NFL-LONG Study. J neurol neurosurg Psychiatry. (2022) 93:272–9. doi: 10.1136/jnnp-2021-326602 PMC8854336 34663623

[31] Gouttebarge V Kerkhoffs G. Sports career-related concussion and mental health symptoms in former elite athletes. Neurochirurgie. (2021) 67:280–2. doi: 10.1016/j.neuchi.2020.01.001 32017942

[32] Bohr AD Boardman JD McQueen MB. Association of adolescent sport participation with cognition and depressive symptoms in early adulthood. Orthop J Sports Med. (2019) 7:2325967119868658. doi: 10.1177/2325967119868658 31598525 PMC6764154

[33] Deshpande SK Hasegawa RB Weiss J Small DS. The association between adolescent football participation and early adulthood depression. PloS One. (2020) 15:e0229978. doi: 10.1371/journal.pone.0229978 32155206 PMC7064245

[34] Iverson GL Merz ZC Terry DP. Playing high school football is not associated with an increased risk for suicidality in early adulthood. Clin J Sport Med. (2021) 31:469–74. doi: 10.1097/JSM.0000000000000890 34704972

[35] Hind K Konerth N Entwistle I Hume P Theadom A Lewis G. Mental health and wellbeing of retired elite and amateur rugby players and non-contact athletes and associations with sports-related concussion: the UK rugby health project. Sports Med. (2022) 52:1419–31. doi: 10.1007/s40279-021-01594-8 PMC9124647 34792798

[36] Baker E Jensen CJ Moeyaert M Bordoff S. Socioeconomic status and early childhood aggression: moderation by theory of mind for relational, but not physical, aggression. Early Child Dev Care. (2020) 190:1187–201. doi: 10.1080/03004430.2018.1524379

[37] Salik I Dominguez JF Vazquez S Ng C Das A Naftchi A. Socioeconomic characteristics of pediatric traumatic brain injury patients. Clin Neurol Neurosurg. (2022) 221:107404. doi: 10.1016/j.clineuro.2022.107404 35987042

[38] Hudson CG. Socioeconomic status and mental illness: tests of the social causation and selection hypotheses. Am J Orthopsych. (2005) 75:3–18. doi: 10.1037/0002-9432.75.1.3 15709846

[39] Macintyre A Ferris D Gonçalves B Quinn N. What has economics got to do with it? The impact of socioeconomic factors on mental health and the case for collective action. Palgrave Commun. (2018) 4:10. doi: 10.1057/s41599-018-0063-2

[40] Reiss F Meyrose AK Otto C Lampert T Klasen F Ravens-Sieberer U. Socioeconomic status, stressful life situations and mental health problems in children and adolescents: Results of the German BELLA cohort-study. PloS One. (2019) 14:e0213700. doi: 10.1371/journal.pone.0213700 30865713 PMC6415852

[41] Zhang Y Su D Chen Y Tan M Chen X. Effect of socioeconomic status on the physical and mental health of the elderly: the mediating effect of social participation. BMC Public Health. (2022) 22:605. doi: 10.1186/s12889-022-13062-7 35351078 PMC8962021

[42] Gaudette E Seabury SA Temkin N Barber J DiGiorgio AM Markowitz AJ. Employment and economic outcomes of participants with mild traumatic brain injury in the TRACK-TBI study. JAMA Netw Open. (2022) 5:e2219444. doi: 10.1001/jamanetworkopen.2022.19444 35767257 PMC9244609

[43] Haines KL Nguyen BP Vatsaas C Alger A Brooks K Agarwal SK. Socioeconomic status affects outcomes after severity-stratified traumatic brain injury. J Surg Res. (2019) 235:131–40. doi: 10.1016/j.jss.2018.09.072 30691786

[44] Spitzer RL Williams JB Gibbon M First MB. The Structured Clinical Interview for DSM-III-R (SCID). I: History, rationale, and description. Arch Gen Psychiatry. (1992) 49:624–9. doi: 10.1001/archpsyc.1992.01820080032005 1637252

[45] Garatachea N Pareja-Galeano H Sanchis-Gomar F Santos-Lozano A Fiuza-Luces C Moran M. Exercise attenuates the major hallmarks of aging. Rejuv Res. (2015) 18:57–89. doi: 10.1089/rej.2014.1623 PMC4340807 25431878

[46] Herbert C. Enhancing mental health, well-being and active lifestyles of university students by means of physical activity and exercise research programs. Front Public Health. (2022) 10:849093. doi: 10.3389/fpubh.2022.849093 35548074 PMC9082407

[47] Asztalos M De Bourdeaudhuij I Cardon G. The relationship between physical activity and mental health varies across activity intensity levels and dimensions of mental health among women and men. Public Health Nutr. (2010) 13:1207–14. doi: 10.1017/S1368980009992825 20018121

[48] Piercy KL Troiano RP Ballard RM Carlson SA Fulton JE Galuska DA. The physical activity guidelines for Americans. JAMA. (2018) 320:2020–8. doi: 10.1001/jama.2018.14854 PMC9582631 30418471

