# Supplementary figures and images for: Associations between repetitive head impact exposure and midlife mental health wellbeing in former amateur athletes
Source: Front Psychiatry. 2024 May 28;15:1383614. doi: 10.3389/fpsyt.2024.1383614 (PMC11165143; doi:10.3389/fpsyt.2024.1383614)

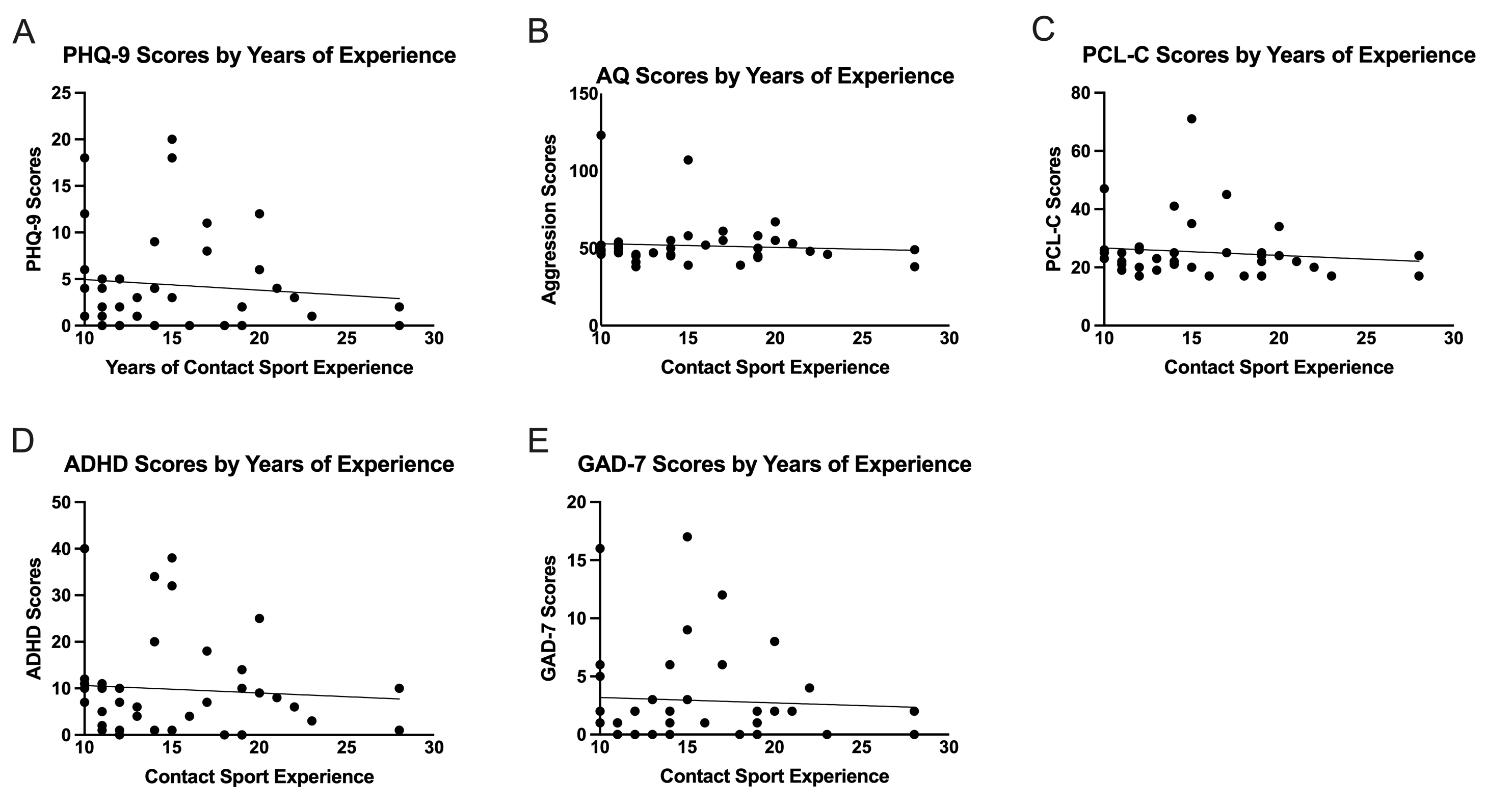

Supplement: Supplementary file 1 [file Image_1.tiff]
