# Supplementary material for: Associations between repetitive head impact exposure and midlife mental health wellbeing in former amateur athletes
Source: Front Psychiatry. 2024 May 28;15:1383614. doi: 10.3389/fpsyt.2024.1383614 (PMC11165143; doi:10.3389/fpsyt.2024.1383614)
Supplement: Supplementary file 2 [file Table_1.docx]

| **Supplemental Table 1: Average mental health scores by group** | | |
| --- | --- | --- |
|  | **Contact Group** | **Non-Contact Control Group** |
| **PHQ-9** | 4.37 (5.23) | 2.00 (3.38) |
| **GAD-7** | 2.95 (4.15) | 1.59 (2.15) |
| **ADHD** | 9.81 (10.69) | 5.27 (7.60) |
| **PCL-C** | 25.34 (10.41) | 20.73 (4.59) |
| **AQ** | 51.73 (15.9) | 47.23 (6.65) |
| *Notes:* average (standard deviation); PHQ-9, depression score; GAD-7, anxiety score; ADHD, attention-deficit hyperactivity disorder; PCL-C, PTSD score; AQ, aggression questionnaire. | | |
