# Supplementary material for: Associations between repetitive head impact exposure and midlife mental health wellbeing in former amateur athletes
Source: Front Psychiatry. 2024 May 28;15:1383614. doi: 10.3389/fpsyt.2024.1383614 (PMC11165143; doi:10.3389/fpsyt.2024.1383614)
Supplement: Supplementary file 3 [file Table_2.docx]

| **Supplemental Table 2:** Average Mental Health Outcome by Income Tier | | | | |
| --- | --- | --- | --- | --- |
| **Contact Group** | | | | |
|  | **$50k and below** | **$51k to $100k** | **$101k - $150k** | **$151k and above** |
| **PHQ-9** | 10.7 (6.3) | 2.14 (2.2) | 2.91 (3.2) | 1.4 (1.3) |
| **GAD-7** | 7.00 (5.8) | 1.29 (1.7) | 2.17 (3.2) | 1.4 (1.7) |
| **ADHD** | 21 (15.0) | 4.71 (4.5) | 6.58 (5.5) | 9.4 (7.33) |
| **PCL-C** | 34.5 (15.6) | 21.85 (3.6) | 23.67 (8.6) | 20.8 (2.6) |
| **AQ** | 65 (27.8) | 47.21 (6.8) | 47.75 (6.1) | 48.8 (2.6) |
| **Non-Contact Control Group** | | | | |
| **PHQ-9** | 1.00 (1.41) | 2.00 (2.0) | 4 (6.7) | 1.43 (2.9) |
| **GAD-7** | 0.23 (0.5) | 1.71 (2.2) | 3.75 (3.0) | 1 (1.4) |
| **ADHD** | 1.25 (2.5) | 6.00 (6.7) | 11.75 (14.2) | 3.14 (3.0) |
| **PCL-C** | 19.5 (4.4) | 21.28 (5.9) | 23.5 (4.5) | 19.28 (3.2) |
| **AQ** | 42.5 (2.5) | 50.57 (8.9) | 51.25 (4.6) | 44.28 (3.6) |
| *Notes:* average (standard deviation); PHQ-9, depression score; GAD-7, anxiety score; ADHD, attention-deficit hyperactivity disorder; PCL-C, PTSD score; AQ, aggression questionnaire. | | | | |
